# Trends in laboratory-confirmed bacterial meningitis (2012–2019): national observational study, England

**DOI:** 10.1016/j.lanepe.2023.100692

**Published:** 2023-07-25

**Authors:** Sathyavani Subbarao, Sonia Ribeiro, Helen Campbell, Ifeanyichukwu Okike, Mary E. Ramsay, Shamez N. Ladhani

**Affiliations:** a Immunisation and Countermeasures Division, UK Health Security Agency, London, UK; b Paediatric Infectious Diseases Research Group (PIDRG), St George's University of London, London, UK; c Department of Infectious Diseases, St George's Hospital, University of London, London, UK; d Department of Paediatrics, Derby Children's Hospital, UK

**Keywords:** Bacterial meningitis, Group B streptococci, Meningococcal meningitis, Pneumococcal meningitis, Surveillance

## Abstract

**Background:**

Bacterial meningitis is associated with significant morbidity and mortality worldwide. We aimed to describe the epidemiology, aetiology, trends over time and outcomes of laboratory-confirmed bacterial meningitis in England during 2012–2019.

**Methods:**

UK Health Security Agency routinely receives electronic notifications of confirmed infections from National Health Service hospital laboratories in England. Data were extracted for positive bacterial cultures, PCR-positive results for *Neisseria meningitidis* or *Streptococcus pneumoniae* from cerebrospinal fluid and positive blood cultures in patients with clinical meningitis.

**Findings:**

During 2012–19, there were 6554 laboratory-confirmed cases. Mean annual incidence was 1.49/100,000, which remained stable throughout the surveillance period (p = 0.745). There were 155 different bacterial species identified, including 68.4% (106/1550) Gram-negative and 31.6% (49/155) Gram-positive bacteria. After excluding coagulase-negative staphylococci (2481/6554, 37.9%), the main pathogens causing meningitis were *Streptococcus pneumoniae* (811/4073, 19.9%), *Neisseria meningitidis* (497/4073, 12.2%), *Staphylococcus aureus* (467/4073, 11.5%), *Escherichia coli* (314/4073, 7.7%) and group B streptococcus (268/4073, 6.6%). Pneumococcal meningitis incidence increased significantly during 2012–9, while meningococcal, group A streptococcal and tuberculous meningitis declined. Infants aged <3 months had the highest mean incidence (55.6/100,000; 95% CI, 47.7–63.5) driven mainly by group B streptococci, followed by 3–11 month-olds (8.1/100,000; 95% CI 7.1–9.0), where pneumococcal and meningitis predominated. The 30-day case-fatality rate (CFR) was 10.0% (71/6554). Group A streptococcal meningitis had the highest CFR (47/85, 55.3%). The probability of surviving at 30 days was 95.3% (95% CI, 93.4–97.3%) for infants and 80.0% for older adults (77–84%).

**Interpretation:**

The incidence of bacterial meningitis has remained stable. The high CFR highlights a need for prevention through vaccination.

**Funding:**

PHE.

**Research in context**

**Evidence before this study**

We searched PubMed using the search term “meningitis” along with “epidemiology”, “incidence”, “trends”, and “rates.” Our search was limited to publications in English since 2010. We searched for population-based surveillance studies in industrialised countries with established national immunisation programmes that provided data for all-cause bacterial meningitis rather than specific pathogens. Nationwide surveillance of cerebrospinal fluid isolates received by the Netherlands reference laboratory for Bacterial meningitis found that overall bacterial incidence had decreased from 6.37 to 1.58 between 1989–1993 and 2014–2019 (IRR, 0.25), with a third of cases occurring in preschool children. In this observational study, the reduction in bacterial meningitis resulted mainly from conjugate vaccines against *Haemophilus influenzae* type b, *Neisseria meningitidis* serogroup C and 10 pneumococcal serotypes. The overall burden of bacterial meningitis was attributed to *Streptococcus pneumoniae*, particularly in older adults and the elderly.

**Added Value of this study**

In this analysis, we updated our previous work on bacterial, mycobacterial and fungal meningitis in England during 2004–11 using the same surveillance and methodology. We found that during 2012–19, the mean annual incidence of bacterial meningitis in England remained stable at 1.49/100,000. The pathogens responsible for bacterial meningitis varied by age, with group B streptococcal meningitis predominating among infants aged <3 months. The incidence of meningococcal meningitis declined while pneumococcal meningitis increased. Overall case-fatality rate was 10%, being higher in children and in older adults aged ≥ 65 years.

**Implications of all the available evidence**

Implementation of new meningococcal vaccines into the national immunisation programme contributed to significant declines in meningococcal meningitis across all age-groups, while the benefits of the pneumococcal immunisation programme appear to have been eroded by serotype-replacement disease. The high case-fatality rates highlight the need for improved preventive measures, including vaccines, against the major bacteria causing meningitis in the different age-groups.

## Introduction

Bacterial meningitis is associated high morbidity and mortality worldwide, with case fatality rate rates ranging from 4% in children and up to 35% in adults).^1^ Survivors are often left with serious long term-sequalae, such as cerebral palsy, epilepsy, blindness and deafness.^2^ The global burden of meningitis has declined by 21% between 1990 and 2016, with cases and deaths disproportionately concentrated within the African meningitis belt.^3^ The aetiology of bacterial meningitis varies by age-group and geography, but vaccinations targeting *H. influenzae*, *N.meningitidis* and *S. pneumoniae* have altered the epidemiological landscape markedly. In the United Kingdom (UK), as in many industrialised countries, *H. influenzae* serotype b (Hib) was one of the leading causes of bacterial meningitis prior to the introduction of the Hib conjugate vaccine into the national childhood immunisation programme in 1992.^4^ Similarly, introduction of the meningococcal group C (MenC) conjugate vaccine in 1999, the infant protein-based meningococcal B (MenB) vaccine in 2015 and the adolescent meningococcal ACWY conjugate vaccine in 215 have led to large and significant declines in invasive meningococcal disease nationally.^5^^,^^6^ In the UK, the 23-valent polysaccharide (PPV) vaccine against *S.pneumoniae*, which has been recommended for older adults and at-risk groups since 1992,^7^ is 41% effective against the vaccine serotypes causing invasive disease within the first 2 years of immunisation.^8^ A 7-valent pneumococcal polysaccharide–protein conjugate vaccine (PCV7) was implemented in the routine childhood immunisation programme in 2006 and replaced with the 13-valent PCV (PCV13) in 2010. Whilst both vaccines led to rapid and sustained declines in invasive pneumococcal disease (including meningitis) caused by the vaccine serotypes,^9^ large increases in cases due to non-vaccine have been observed in recent years.^10^

There are very few population-based surveillance data for clinical syndromes such as meningitis, with most publications focussing on specific pathogens or immunisation programmes. We have previously reported trends in meningitis using national laboratory surveillance data for England during 2004–11, when annual incidence remained stable overall and across the age-groups, apart from significant year-on-year increases among infants aged <3 months, driven mainly by group B streptococci (GBS), and in adults aged ≥65 years, primarily because of *Escherichia coli.*^11^ Here, we use national laboratory surveillance to describe the epidemiology, trends over time and case-fatality rates associated with bacterial meningitis in England during 2012–19, prior to the COVID-19 pandemic.

## Methods

The UK National Health Service (NHS) hospital laboratories report all confirmed infections electronically to UK Health Security Agency (UKHSA) using the Second Generation Surveillance System (SGSS). Records of all positive bacterial cultures, PCR positivity, antibody/antigen detection from the cerebrospinal fluid (CSF), or from blood in patients recorded to have a clinical diagnosis of bacterial meningitis,^12^ during 2012–2019 were extracted from SGSS, cleaned, and de-duplicated for analysis. Unique individual NHS numbers, last name, first name, sex and date of birth were used to link with the patient demographic service (PDS), a national electronic database containing demographic details of all individuals registered with the NHS, to determine date of death. Isolation of the same pathogen within 30 days was considered a single episode. Reports with more than one pathogen from the same sample were analysed separately. SGSS does not provide clinical data for reported cases and, bacteria considered to be environmental or skin contaminants except for Coagulase negative staphylococci, were excluded from (Appendix) the analysis.

### Statistical analysis

Data were analysed using R version 4.0.1. Age-specific incidence rates were calculated using mid-year resident population estimates obtained from the UK Office for National Statistics. Standard linear regression was used to calculate changes in annual incidence rates with 95% confidence intervals (CI). Deaths occurring within 30 days of laboratory confirmation or significant isolates reported within 30 days after the date of death (most likely during post-mortem investigations) were used to calculate the case-fatality rate (CFR). Categorical variables are expressed as proportions and logistic regression was used to calculate odds-ratio for death in univariable and multivariable analysis. The incidence rate ratio (IRR) with 95% CI were used to compare incidence rates by age, sex and other parameters. We did not correct for multiple comparisons in our analysis, which means that associations with borderline statistical significance around p values of 0.05 may be due to chance.

Kaplan–Meier analysis was used to compare survival between different groups. We restricted follow-up period to 30 days such that those that did not link to PDS (and, therefore, their outcome was unknown) and those that died >30 days after their test date were censored in the Kaplan–Meier survival curves.

### Ethics approval

UKHSA has legal permission, provided by Regulation 3 of The Health Service (Control of Patient Information) Regulations 2002, to process patient confidential information for national surveillance of communicable diseases and as such, individual patient consent is not required.

### Role of the funding source

There was no funding source for this study. The corresponding author had full access to all the data in the study and had final responsibility for the decision to submit for publication.

## Results

During 2012–19, a total of 6554 individuals had laboratory-confirmed meningitis in England. A diagnosis by culture was made in 4687/6554 (71.5%) and PCR 388/6554 (5.9%). Of the total, 47.7% (3125/6554) were female (mean incidence 1.39, 95% CI 1.31–1.48) and 52.3% (3429/6554%) were male (mean incidence 1.59, 95% CI 1.49–1.70); IRR 0.87 (95% CI 0.76–1.01, p = 0.06). Mean annual incidence was 1.49/100,000 and remained stable through the period (p = 0.745). In total, 155 different bacterial species were isolated and 68.4% (106/1550) were Gram-negative while 31.6% (49/155) were Gram-positive bacteria (Table 1).

**Table 1:** National incidence per 100,000 of laboratory-confirmed bacterial meningitis due to specific pathogens by calendar year in England and trends over time.

Pathogen	2012	2013	2014	2015	2016	2017	2018	2019	Mean incidence (mean annual total)/100,000	Change in incidence per year/100,000 (95% CI)	p value
	Incidence (N)	Incidence (N)	Incidence (N)	Incidence (N)	Incidence (N)	Incidence (N)	Incidence (N)	Incidence (N)			
Enterococcus sp.	0.04 (23)	0.04 (24)	0.07 (38)	0.04 (20)	0.04 (21)	0.03 (14)	0.04 (21)	0.05 (27)	0.04 (23.5)	−0.001 (−0.005, 0.004)	0.772
*L. monocytogenes*	0.02(11)	0.02 (13)	0.03 (14)	0.02 (12)	0.01 (6)	0.02 (10)	0.02 (12)	0.01 (5)	0.02 (10.38)	−0.001 (−0.004, 0.001)	0.206
*S. aureus*	0.1 (56)	0.1 (54)	0.11 (58)	0.1 (57)	0.1 (58)	0.12 (64)	0.11 (59)	0.11 (61)	0.11 (58.38)	0.002 (−0.001, 0.004)	0.125
*S. pneumoniae*	0.17 (89)	0.15 (79)	0.17 (90)	0.19 (106)	0.2 (110)	0.2 (112)	0.19 (108)	0.21 (117)	0.18 (101.38)	0.007 (0.003, 0.011)	0.008
Group A Streptococcus	0.03 (17)	0.04 (22)	0.02 (10)	0.02 (11)	0.01 (4)	0.02 (10)	0.01 (7)	0.01 (4)	0.02 (10.62)	−0.004 (−0.006, −0.001)	0.013
Group B Streptococcus	0.05 (26)	0.05 (25)	0.08 (43)	0.07 (38)	0.05 (25)	0.06 (33)	0.08 (42)	0.06 (36)	0.06 (33.5)	0.002 (−0.003, 0.007)	0.442
Other Gram Pos	0.05 (25)	0.04 (23)	0.05 (25)	0.04 (24)	0.04 (20)	0.02 (13)	0.05 (30)	0.04 (21)	0.04 (22.62)	−0.001 (−0.005, 0.003)	0.434
Enterobacter sp.	0.04 (21)	0.03 (14)	0.03 (15)	0.03 (14)	0.03 (18)	0.04 (20)	0.03 (15)	0.03 (17)	0.03 (16.75)	0 (−0.002, 0.001)	0.547
*E. coli*	0.12 (62)	0.07 (40)	0.08 (43)	0.05 (30)	0.06 (32)	0.07 (41)	0.06 (33)	0.06 (33)	0.07 (39.25)	−0.006 (−0.012, 0.001)	0.075
*H. influenzae*	0.02 (9)	0.01 (7)	0.02 (10)	0.02 (13)	0.02 (13)	0.02 (10)	0.03 (14)	0.02 (13)	0.02 (11.12)	0.001 (−0.001, 0.003)	0.162
Klebsiella sp.	0.05 (28)	0.03 (17)	0.05 (26)	0.02 (13)	0.04 (20)	0.04 (24)	0.04 (20)	0.04 (25)	0.04 (21.62)	0 (−0.004, 0.004)	0.835
*N. meningitidis*	0.17 (89)	0.14 (74)	0.1 (57)	0.13 (71)	0.1 (56)	0.1 (58)	0.09 (50)	0.07 (42)	0.11 (62.12)	−0.012 (−0.018, −0.006)	0.003
Pseudomonas sp.	0.04 (24)	0.04 (21)	0.03 (15)	0.05 (25)	0.05 (28)	0.03 (19)	0.05 (30)	0.04 (21)	0.04 (22.88)	0.001 (−0.003, 0.004)	0.679
Other Gram Neg	0.13 (68)	0.09 (51)	0.11 (59)	0.06 (32)	0.09 (48)	0.09 (48)	0.08 (44)	0.09 (49)	0.09 (49.88)	−0.004 (−0.011, 0.003)	0.195
CNS	0.6 (323)	0.59 (320)	0.55 (297)	0.47 (258)	0.56 (309)	0.61 (342)	0.62 (346)	0.51 (286)	0.56 (310.12)	−0.003 (−0.024, 0.019)	0.783
*M. tuberculosis*	0.08 (44)	0.04 (24)	0.04 (22)	0.05 (25)	0.05 (28)	0.04 (24)	0.03 (15)	0.03 (19)	0.04 (25.12)	−0.005 (−0.009, 0)	0.041
Total	1.71 (915)	1.5 (808)	1.51 (822)	1.37 (749)	1.44 (796)	1.51 (842)	1.51 (846)	1.38 (776)	1.49 (819.25)	**−**0.026(**−**0.061, 0.009)	0.118

Abbreviations: L.monocytogenes = Listeria monocytogenes; S.aureus = Staphylococcus aureus, S.pneumoniae = Streptococcal pneumoniae, E.coli = Escherichia coli, H.influenzae-Haemophilus influenzae, N.meningitidis = Neisseria meningitidis, CNS= Coagulase negative Staphylococci, M.tuberculosis = Mycobacterium tuberculosis).

Coagulase-negative staphylococci (CNS) accounted for 37.9% (2481/6554) of reported cases. Following exclusion of CNS, the main pathogens were *Streptococcus pneumoniae* (811/4073, 19.9%), *Neisseria meningitidis* (497/4073, 12.2%), *Staphylococcus aureus*, 467/4073 (11.5%), *Escherichia coli* (314/4073, 7.7%), *group B Streptococcus* (GBS) (268/4073, 6.6%). *Mycobacterium tuberculosis* (201/4073, 4.9%). *Enterococcal* species (188/4073, 4.6%) *Pseudomonas aeruginosa* (183/4073, 4.5%), *Klebsiella pneumoniae* (173/4073, 4.3%), *Haemophilus influenzae* (89/4073, 2.2%) and group A Streptococci (GAS) (85/4073, 2.1%) constituted the remaining main pathogens causing meningitis in England.

**Fig. 1:**
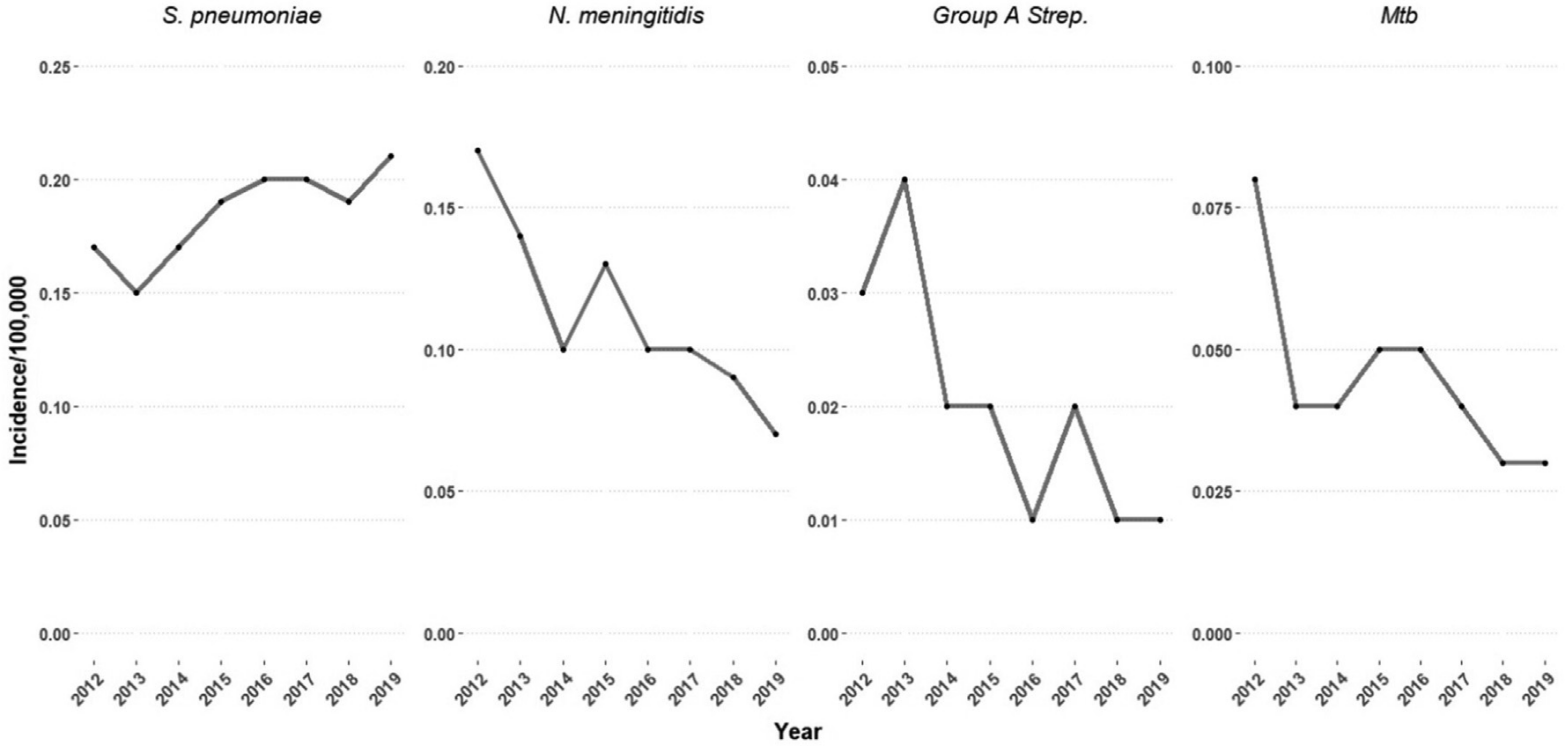
Trends in annual incidence of bacterial meningitis per 100,000 population due to bacteria with a significant change over time during 2012–19. Abbreviations: S.pneumoniae = Streptococcal pneumoniae, N.meningitidis = Neisseria meningitidis, M.tuberculosis = Mycobacterium tuberculosis.

Seasonal variation among Gram-positive bacteria was observed with *S pneumoniae*, which increased during the winter months, and GAS, which exhibits a bimodal peak during Spring and Winter (Supplementary Figure S1). Among Gram-negative pathogens, *N. meningitidis* and *H. influenzae* also exhibited a winter peak, with a fall in the summer months. When assessing trends over time, the incidence of pneumococcal meningitis increased during 2012–2019, while GAS, *Mtb* and *N. meningitidis* declined, although there was a small incidence peak in meningococcal incidence in 2015 (Fig. 1).

### Age and pathogens

**Fig. 2:**
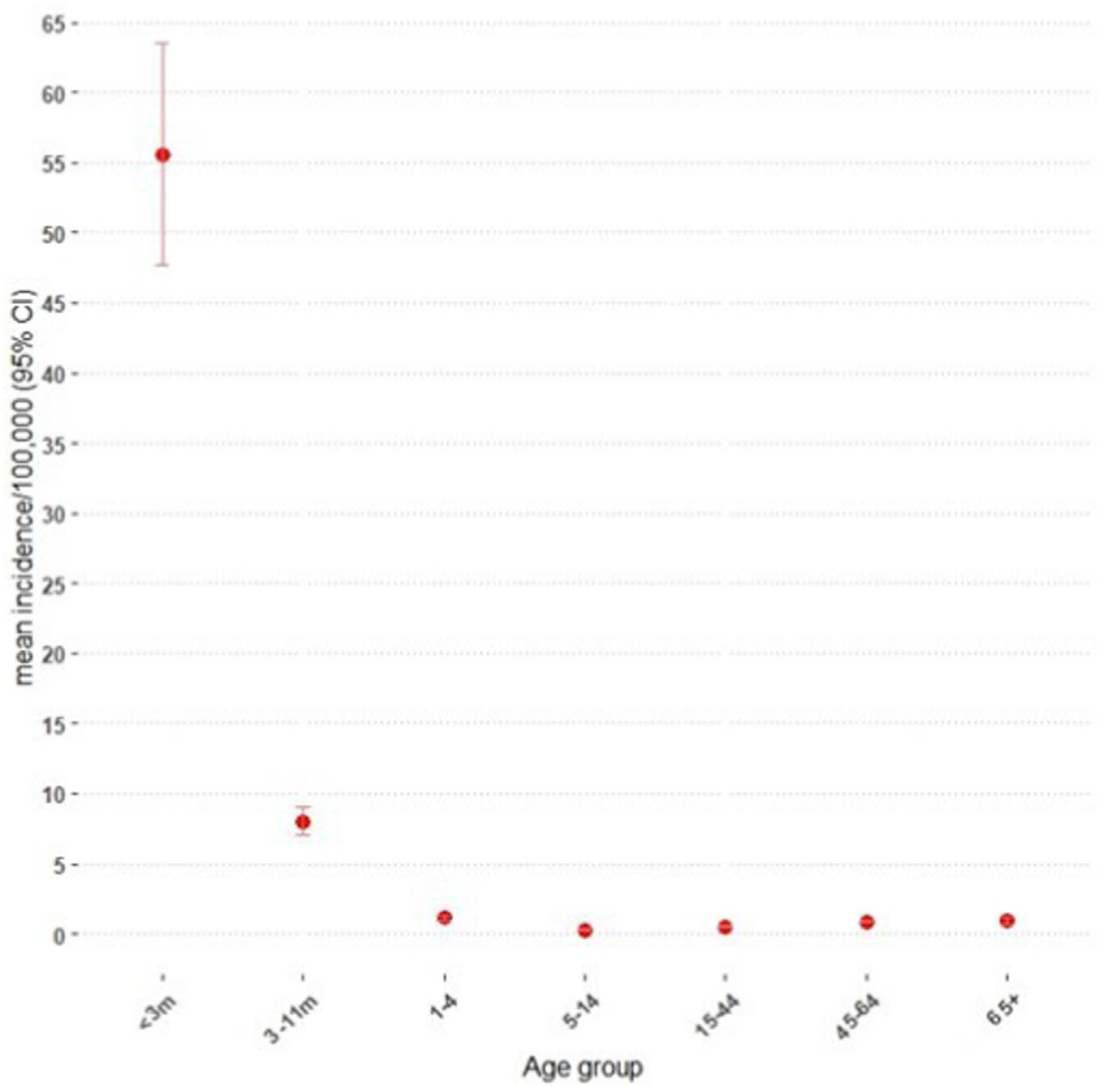
Mean annual incidence of bacterial meningitis cases per 100,000 with 95% confidence intervals by age group in England during 2012–2019.

The age distribution of bacterial meningitis cases included 20.0% (732/4073) in <3 months, 7.8% (319/4073) in 3–11 months, 6.1% in 1–4 year olds (249/4073), 3.6% in 5–14 year olds (146/4073), 21.5% (875/4073) in 15–44 year-olds, 24% (977/4073) in 45–64 year-olds and 18.7% (763/4073) in ≥ 65 year-olds. Infants aged <3 months had the highest mean incidence (55.6/100,000; 95% CI, 47.7–63.5) followed by 3–11 month-olds (8.1/100,000; 95% CI 7.1–9.0) (Fig. 2).

The contribution of the different bacteria to meningitis varied with age (Supplementary Figure S2). In <3 month-olds, GBS (230/732, 31.4%), *E. coli* (107/732, 14.6%), *N. meningitidis* (43/732, 5.9%), *S. aureus* (41/732, 5.6%) *and S. pneumoniae* (37/732, 51%) comprised the top five pathogens. In this age-group, 78/230 (33.9%) GBS cases were early onset (within 7 days of birth). Among 3 month to 14 year-olds, *N. meningitidis* (225/714, 31.5%), *S. pneumoniae* (174/714, 24.4%), *S. aureus (*71/714, 10.0%), GAS (41/714, 5.6%) and *E. coli (*34/714, 4.8%) were the top five pathogens. In adults aged 15–64 years, the top five pathogens were *S. pneumoniae (*384/1852, 20.7%), *S. aureus* (287/1852, 15.5%), *N. meningitidis* (197/1852, 10.6%), *Mtb* (160/1852, 8.7%) and *E. coli* (104/1852, 5.7%). Among older adults aged ≥65 years, *S. pneumoniae (*213/763, 27.9%) was the main pathogen, followed by *E. coli* (69/763, 9.0%), *S. aureus* (68/763, 8.9%), *P. aeruginosa* (37/763, 4.9%) and *L. monocytogenes* (32/763, 4.2%).

### Incidence trends by age

**Fig. 3:**
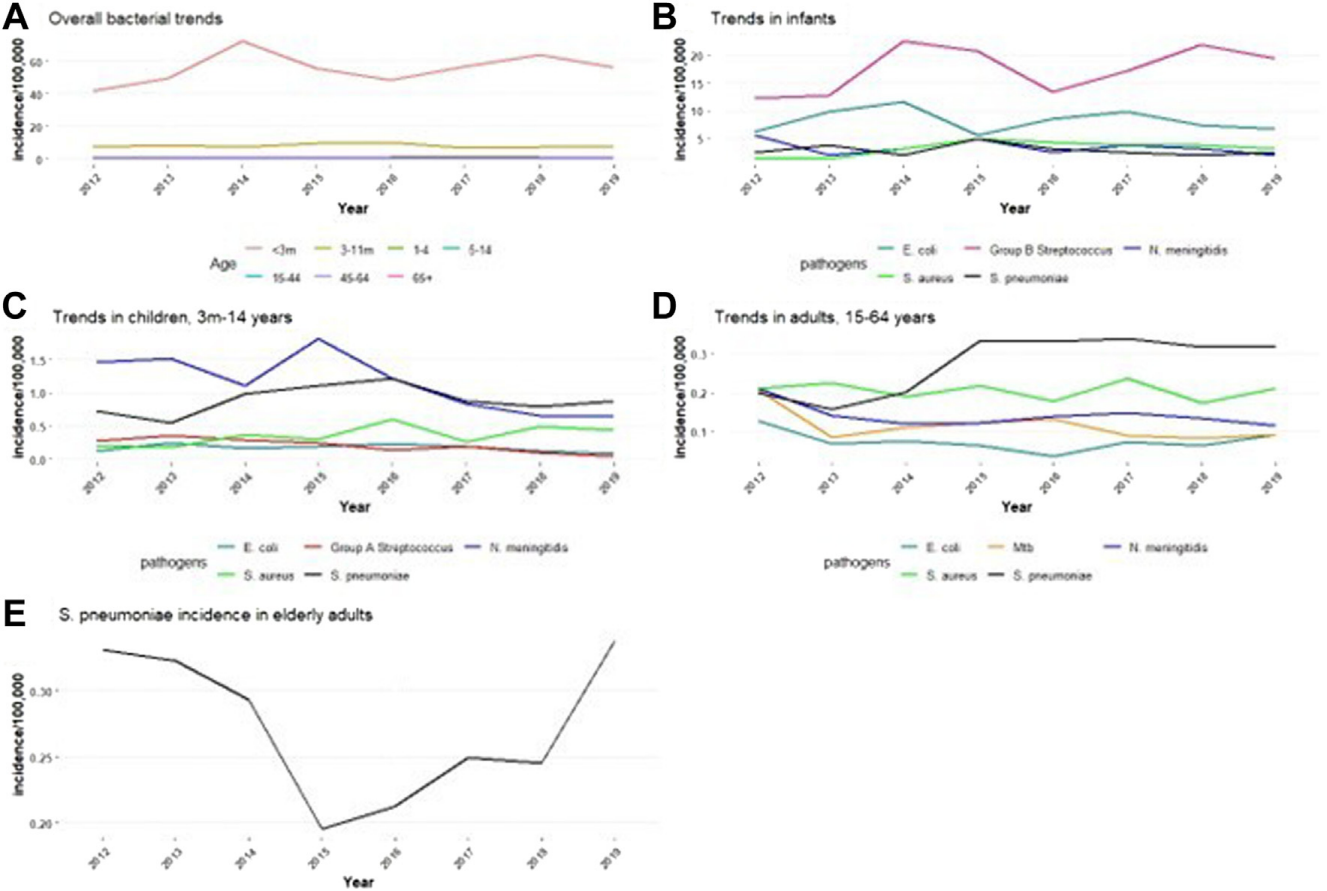
Trends in incidence per 100,000 of bacterial meningitis by age group in England during 2012–19. A: Overall incidence by age group. B Incidence per 100,000 for the overall top five pathogens in infants aged <3 months. C. Incidence per 100,000 for the overall top five pathogens in children aged 3 months to 14 years. D. Incidence per 100,000 for the overall top five pathogens in adults aged 15–64 years. E: Incidence per 100,000 for pneumococcal meningitis in older adults aged 65+ years. Note the different axis scales. Abbreviations: S.aureus = Staphylococcus aureus, S.pneumoniae = Streptococcal pneumoniae, E.coli = Escherichia coli, N.meningitidis = Neisseria meningitidis, M.tuberculosis = Mycobacterium tuberculosis. Trends only shown in bacteria where total numbers exceed 50.

During 2012–2019, the overall incidence of bacterial meningitis was stable for all age groups (Supplementary Table S1). The highest incidence was in <3 month-olds, where the incidence ranged from 41.3/100,000 in 2012 to 72.3/100,000 in 2014 (Fig. 3A), reflecting trends in GBS meningitis incidence (Fig. 3B). To examine annual trends in specific pathogens over time, cases in children aged 3–11 months, 1–4 years and 5–14 years were pooled together and showed significant declines in the incidence of GAS meningitis (0.04/100,000 per year; 95% CI −0.06 to 0.02/100,000; p < 0.001) meningococcal meningitis (0.14/100,000 per year; 95% CI, −0.24 to −0.03/100,000; p < 0.05) (Fig. 3C). Among adults aged 15–64 years, annual *Mtb* meningitis incidence declined by 0.01 per year but this was borderline statistically significant (p = 0.05), while annual pneumococcal meningitis incidence increased by 0.02/100,000 per year (95% CI, 0.01–0.04/100,000, p = 0.02) (Fig. 3D). Among older adults, only pneumococcal meningitis was assessed because of small numbers and there were no significant trends (p = 0.57) although there were incidence peaks in 2012 (0.33/100,000) and 2019 (0.34/100,000) (Fig. 3E).

### Case fatality rate

The overall 30-day CFR was 10.0% (671/6554) and this was similar among females (314/3125, 10.0%) and males (357/3429, 10.4%). By age-group, the CFR was 9.3% (111/1198) in <3 month-olds, 14.9% (128/923) in 3 month–14 year-olds, 7.2% (233/3226) in 15–64 year-olds and 16.7% (199/1193%) in ≥65 year-olds (Supplementary Figure S3). Although overall numbers of GAS meningitis cases were low, they were associated with the highest CFR (38/85, 44.7%) followed by *S. pneumoniae* (134/811, 16.5%) and *E. coli*. (49/314, 15.6%) (Supplementary Figure S3).

In the univariate analysis, children aged 3 months to 14 years, older adults aged ≥65 years, *S. aureus*, *E. coli*, *S. pneumoniae*, GAS, GBS and *Mtb* had an increased odds of death at 30 days in unadjusted analysis and these were all independently associated with death in the logistic regression model (Table 2), with the highest odds of death associated with GAS meningitis (26.0; 95% CI 13-5-52.4; p < 0.001).

**Table 2:** Univariable and multivariable analysis comparing fatal cases and survivors of bacterial meningitis 30 days after diagnosis.

Variable	Alive	Dead	CFR(%)	Univariable			Multivariable		
				OR	p value	95% CI	OR	p value	95% CI
Total	**3738**	**507**	**11.9**						
Female	1774	233	11.6	–	–	–	–	–	–
Male	1964	274	12.2	1.06	0.53	(0.88, 1.28)	1.08	0.45	(0.89, 1.3)
Adults	1764	185	9.5	–	–	–	–	–	–
Infants	710	66	8.5	0.89	<0.001	(0.66, 1.18)	0.82	0.25	(0.58, 1.14)
Children	627	103	14.1	1.57	<0.001	(1.21, 202)	1.66	<0.001	(1.25, 2.19)
Elderly	637	153	19.4	2.29	<0.001	(1.81, 2.89)	2.12	<0.001	(1.67, 2.68)
*N. meningitidis*	481	15	3	–	–	–	–	–	–
*S. aureus*	432	35	7.5	2.6	<0.001	(1.43, 4.96)	2.84	<0.001	(1.54, 5.46)
*S. pneumoniae*	674	134	16.6	6.38	<0.001	(3.81, 11.46)	6.16	<0.001	(3.65, 11.15)
*Group A Streptococcus*	47	38	44.7	25.93	<0.001	(13.55, 51.97)	26	<0.001	(13.52, 52.35)
*Group B Streptococcus*	241	25	9.4	3.33	<0.001	(1.74, 6.57)	5.04	<0.001	(2.51, 10.43)
*E. coli*	265	49	15.6	5.93	<0.001	(3.34, 11.13)	6.56	<0.001	(3.64, 12.47)
*Mtb*	170	29	14.6	5.47	<0.001	(2.91, 10.71)	6.28	<0.001	(3.28, 12.48)
*Other*	14,281	182	11.3	4.09	<0.001	(2.47, 7.29)	4.37	<0.001	(2.61, 7.88)

OR = odds ratio. CI=Confidence interval.

### Survival analysis

There was no statistical difference in 30-day survival rates between males and females but there was a significant difference between the four age groups including infants, children, adults and older adults (Supplementary Figure S4). The probability of survival at 30 days for infants was 95.3% (95% CI 93.4–97.3) and 80.0% for elderly (77–84%). Similarly, 30-day survival rates varied for the different pathogens, being 97.8% (96.5–99.1%) for *N. meningitidis*, compared to 85.7% (83.3–88.2%) for *S. pneumoniae* and 85.5% (76.7–95.3%) for GAS.

## Discussion

In England, laboratory-based national surveillance identified stable rates of bacterial meningitis during 2012–2019. Incidence remained highest in infants aged <3 months, driven mainly by GBS, followed by 3–11 month-olds, where *N. meningitis* and *S. pneumoniae* were the main pathogens responsible. Trends in specific pathogens identified significant declines in meningococcal, GAS and Mtb meningitis rates, but an increasing trend in pneumococcal meningitis rates. We have for the first time reported 30-day CFR by age and pathogen, and identified a high CFR associated specifically with GAS meningitis. In a logistic regression model, a fatal outcome was independently associated with the youngest and oldest age groups compared to adults and with meningitis due to *S. aureus*, GAS, GBS, *E. coli* and *Mtb* compared to meningitis due to *N. meningitis*.

In our previous analysis during 2004–11, we had reported large declines in meningococcal meningitis cases over time, due to the success of the MenC immunisation programme,^6^ as well as secular declines in MenB cases.^11^ A national outbreak of group W meningococcal disease (MenW) belonging to the ST-11 clonal complex, which began in 2009/10, led to emergency implementation of a national adolescent immunisation programme with the MenACWY conjugate vaccine in August 2015 to provide direct protection for vaccinated teenagers and, because conjugate vaccines also prevent nasopharyngeal carriage and interrupt onward transmission, provide indirect (herd) protection across the population.^13^ Notably, a school-based MenACWY vaccine programme for younger adolescents achieved very high vaccine uptake compared to a primary-care based MenACWY immunisation programme for 18 year-olds, thus ensuring better long-term population protection in the coming years^14^

At the same time, a newly-licensed, broad-spectrum, protein-based MenB vaccine (4CMenB) was introduced into the national infant immunisation programme in September 2015. Like all the other routine vaccines in the infant immunisation programme, 4CMenB uptake was very high, with >90% of infants receiving their vaccine in a timely manner.^15^ Although 4CMenB is licensed for preventing MenB disease, the protein antigens in the vaccine are found on the surface of all meningococci and, therefore, could provide broader protection against all meningococci in vaccinated individuals.^16^ In England, whilst 4CMenB only provided direct protection to vaccinated children, the indirect impact of the adolescent MenACWY immunisation programme led to significant overall declines in meningococcal disease cases, especially due to MenW and MenY in subsequent years.^5^^,^^17^^,^^18^ This is reflected in the overall trends in meningococcal meningitis in our current analysis.

In addition to *N. meningitidis*, *S. pneumoniae* is also a major cause of bacterial meningitis. There are currently >100 known pneumococcal serotypes and currently-licensed conjugate vaccines protect against only 13 of the most common serotypes causing IPD. PPV23 aims to protect against 23 serotypes but, because it is polysaccharide-only vaccine, protection is short-lived, with lower vaccine effectiveness compared to conjugate vaccines, and, since polysaccharide vaccines do not affect carriage, there is no indirect (herd) protection. In England, PCVs have led to large and sustained reductions in IPD due to the vaccine serotypes (including meningitis) across all age-groups.^10^^,^^19^ PCV7-type and PCV13-type IPD had declined by 97% and 64%, respectively, by 2016/17.^10^ The decline in carriage and IPD due to the vaccine serotypes, however, was soon followed by replacement with non-vaccine serotypes, initially in carriage and then in disease, which subsequently led to an increase in IPD cases nationally.^10^^,^^20^ Although reports of pneumococcal meningitis through SGSS does not contain any serotype information, this increase reflects national trends in serotype-replacement IPD, which has disproportionately affected adults and older adults compared to children.^10^ Some of these replacing serotypes, such as serotype 8, have a higher propensity to cause meningitis compared to septicaemia and have a higher CFR when compared to other serotypes.^21^

An unexpected finding, even when compared to the previous analysis, was the increase in GAS meningitis which was associated with the highest CFR in the current analysis. Invasive GAS (iGAS) is a statutorily notifiable disease and is temporally associated with scarlet fever, another notifiable disease. In England, cases of scarlet fever and iGAS rose in 2014 and peaked in 2016, which coincided with the peaks in GAS meningitis during 2013 and 2017.^22^ Fortunately, GAS meningitis remains rare; there were 85 cases during 2012–19, with almost half the cases diagnosed in young children. In contrast, *Mtb* meningitis cases declined during the surveillance period, mainly among adults, which is consistent with national data reporting year-on-year incidence reduction in overall *Mtb* rates, except for a small rise in cases during 2019.^23^

When analysed by age-group, infants and particularly <3 month-olds have consistently been disproportionately affected by bacterial meningitis, with GBS being responsible for almost a third of cases. GBS is a transient coloniser of the genital and gastrointestinal tracts in a third of pregnant women and maternal-infant transmission usually occurs prior to or during childbirth.^24^ Unlike other countries with universal screening for GBS antenatally, the UK has opted for selective screening of high-risk pregnancy only. National surveillance indicates that GBS incidence in young infants has been increasing,^25^ which is consistent with our current analysis for GBS meningitis. Antenatal screening for GBS, however, has limited impact on late-onset GBS disease, including meningitis,^26^ highlighting a need for antenatal vaccination to prevent both early-onset and late-onset GBS disease.

Bacterial meningitis is associated with a poor prognosis, both in terms of mortality and long-term morbidity among survivors.^27^ Worldwide, the burden of death due to meningitis has reduced due to a combination of vaccination, antibiotics and steroids where appropriate,^28^ but the highest mortality remains across the meningitis belt,^3^ where large epidemics of meningococcal disease, including meningitis, occur frequently.^29^ In the UK, the national institute for clinical excellence (NICE) provides evidence-based guidance with regular updates on the investigation, diagnosis and management of bacterial meningitis in adults and children.^1^^,^^30^ With the current analysis, we have for the first time reported 30-day CFR in individuals with laboratory-confirmed bacterial meningitis. The overall CFR was 10%, with the highest odd of death in children and the elderly when compared to adults. This differs to the global situation, where death rates are highest <5 year-old, especially neonates,^3^ likely due to healthcare disparities and access to routine childhood vaccines in low and middle income settings. The higher CFR among older adults with bacterial meningitis, as well as a clear difference in 30-day survival curves compared to the other age-groups, is consistent with reported studies high income countries.^31^^,^^32^ It is likely that our meningitis rates in older adults are underestimated because they may be more likely to have atypical clinical presentations and less likely to have lumbar punctures performed. Using meningococcal meningitis as a reference baseline, we found a higher CFR for pneumococcal, GAS, GBS, *E. coli* and *Mtb* meningitis, which is consistent with the global picture, where non-meningococcal meningitis contributes to a higher proportion of meningitis deaths. CFR following pneumococcal meningitis was 16.5% which is consistent with our national surveillance data.^21^ Although GAS meningitis was rare, the 44% CFR was very high, with 26 times the odds of death compared to meningococcal meningitis. In Netherlands, an observational study of adults with GAS meningitis reported a CFR of 19% (5/26 cases)^33^ but CFR in a population study of Brazilian children was 43%.^34^

There are limitations to our study. Firstly, our surveillance only included laboratory-confirmed cases where a pathogen was isolated. Therefore, culture-negative (and PCR-negative for pneumococcal and meningococcal meningitis) cases were excluded, even if local CSF findings might have been consistent with bacterial meningitis on microscopy, cellular, protein and glucose analysis. Clinically diagnosed meningitis cases were also excluded unless a pathogen was isolated in the blood and meningitis was recorded in the SGSS report. Our estimates must, therefore, be regarded as minimum incidence but the pathogens responsible and trends over time should be representative of the population. Secondly, we excluded potential contaminating bacteria because they rarely cause meningitis in immunocompetent hosts and we had no clinical data, such as immune status or presence of intraventricular shunts, to support or refute the diagnosis of bacterial meningitis or similarly whether this was a post-surgical meningitis. This was particularly the case for CoNS which is a common skin contaminant, but can cause meningitis in those with CSF shunts, for example see Azimi et al.^35^ Because of a lack of clinical data, we also did not have any information on underlying comorbidities, which would have been valuable for assessing risk factors for specific organisms, age-groups, nosocomial versus community-acquired infections and outcomes of bacterial meningitis.

In conclusion, this study provides valuable population-based analysis of laboratory-confirmed bacterial meningitis, with trends and CFR by age-group and pathogen over a period of nearly a decade. Whist the overall incidence remained stable, we found significant changes in the epidemiology of bacterial meningitis compared to the previous decade, particularly the reduction in meningococcal meningitis because of a comprehensive meningococcal immunisation programme in England. On the other hand, the increasing incidence of pneumococcal meningitis highlights the plight of replacing serotypes following PCV implementation and demonstrates the need for higher-valent PCVs at least until a serotype-independent universal vaccine becomes available.^36^ We also observed important trends in pathogens causing meningitis that are as yet not preventable through vaccination. In particular, there is an urgent need for an antenatal GBS vaccine to protect young infants. The high overall CFR of 10% and a higher burden of long-term neuro-developmental complications among survivors of bacterial meningitis emphasises the importance of prevention through vaccination.

## Contributors

SS wrote the original draft, performed the formal analysis, methodology and visualisation; SR did the data collection and curation; HC and IO helped with writing the manuscript, methodology and supervision; MR helped write the manuscript and supervision; SNL conceived the idea, reviewed and edited the manuscript, provided support with the methodology and supervised the study overall.

## Declaration of interests

The authors declare no conflicts of interest.

## Appendix A Supplementary data

Supplementary Section
