# Supplementary material for: Trends in laboratory-confirmed bacterial meningitis (2012–2019): national observational study, England
Source: Lancet Reg Health Eur. 2023 Jul 25;32:100692. doi: 10.1016/j.lanepe.2023.100692 (PMC10393823; doi:10.1016/j.lanepe.2023.100692)
Supplement: Supplementary Section [file mmc1.docx]

**SUPPLEMENTARY**

**DOCUMENT**

**Trends in laboratory-confirmed bacterial meningitis (2012-2019): national observational study, England**

Sathyavani Subbarao^1,3^, Sonia Ribeiro^1^, , Helen Campbell,^1^ Ifeanyichukwu Okike^4^, Mary E Ramsay^1^, Shamez N Ladhani,^1,2^

|  | 2012 | 2013 | 2014 | 2015 | 2016 | 2017 | 2018 | 2019 | mean incidence(mean annual total)/100,000 | change in incidence per year/100,000 (95% C.I) | p value |
| --- | --- | --- | --- | --- | --- | --- | --- | --- | --- | --- | --- |
| Age group | Incidence (N) | Incidence (N) | Incidence (N) | Incidence (N) | Incidence (N) | Incidence (N) | Incidence (N) | Incidence (N) |  |  |  |
| <3m | 41.93 (73) | 49.67 (84) | 72.27 (120) | 55.51 (92) | 48.42 (81) | 56.93 (93) | 63.97 (102) | 56.23 (87) | 55.61 (732) | 1.411(-2.171,4.993) | 0.372 |
| 3-11m | 7.47 (39) | 8.28 (42) | 7.43 (37) | 9.45 (47) | 10.16 (51) | 6.73 (33) | 7.32 (35) | 7.54 (35) | 8.05 (319) | -0.067(-0.544,0.409) | 0.741 |
| 1-4 | 1.11 (30) | 1.06 (29) | 1.48 (41) | 1.01 (28) | 1.09 (30) | 1.1 (30) | 1.26 (34) | 1.01 (27) | 1.14 (249) | -0.01(-0.074,0.054) | 0.718 |
| 5-14 | 0.31 (19) | 0.45 (28) | 0.24 (15) | 0.17 (11) | 0.23 (15) | 0.26 (17) | 0.38 (26) | 0.22 (15) | 0.28 (146) | -0.011(-0.048,0.026) | 0.495 |
| 15-44 | 0.67 (143) | 0.52 (111) | 0.5 (106) | 0.47 (100) | 0.49 (104) | 0.55 (117) | 0.42 (90) | 0.49 (104) | 0.51 (875) | -0.019(-0.042,0.004) | 0.096 |
| 45-64 | 1.02 (138) | 0.71 (96) | 0.82 (113) | 0.97 (135) | 0.86 (121) | 0.84 (119) | 0.87 (124) | 0.91 (131) | 0.87 (977) | -0.001(-0.04,0.039) | 0.967 |
| 65+ | 1.58 (143) | 1.04 (97) | 0.98 (93) | 0.8 (78) | 0.84 (83) | 0.9 (90) | 0.86 (88) | 0.88 (91) | 0.99 (763) | -0.071(-0.145,0.003) | 0.057 |

Supplementary Table 1: Incidence of laboaratory-confirmed bacterial meningitis by age-group and year, with trends over time and 95% confidence interval estimated by linear regression analysis.


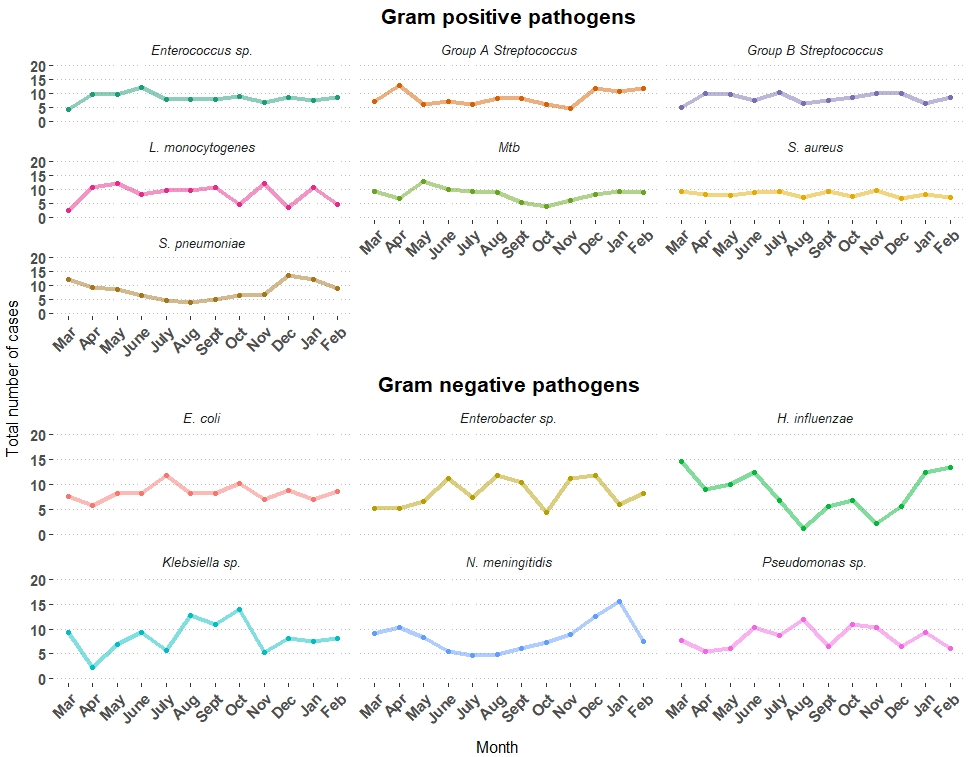


Supplementary Figure 1. Total numbers of cases of laboratory-confirmed bacterial meningitis by month of year for the major Gram positive and Gram-negative pathogens.


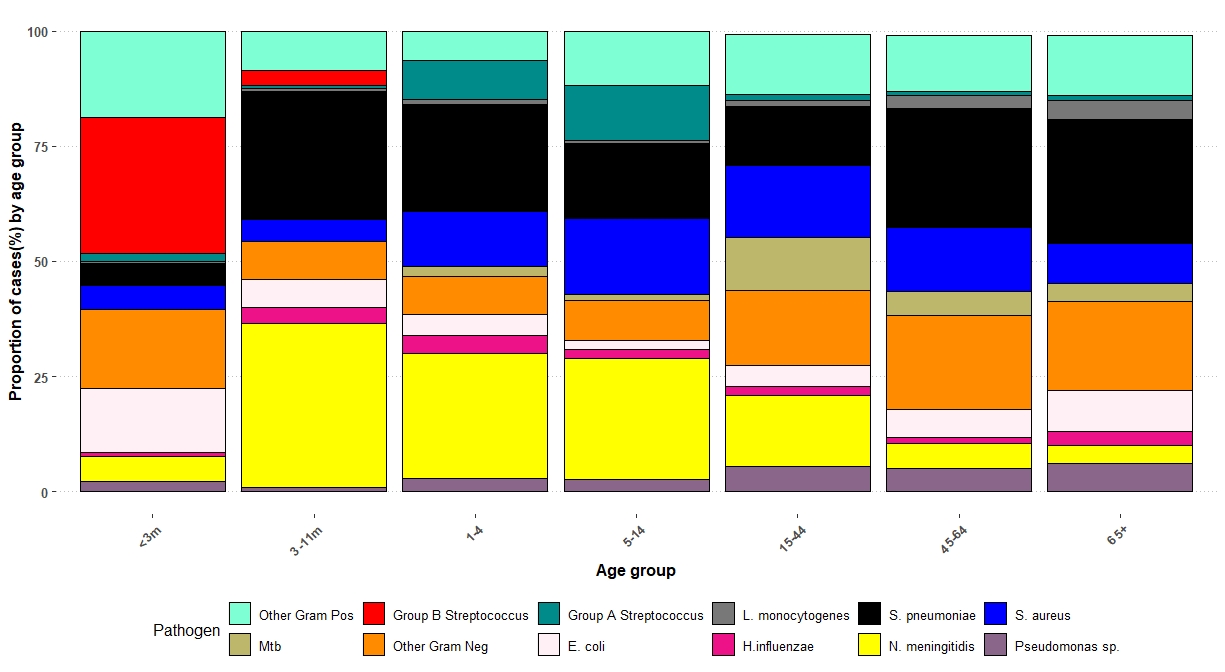


Supplementary Figure 2. Proportions of bacterial meningitis cases caused by specific bacteria in different age groups during 2012-19 in England. Abbreviations: L.monocytogenes= Listeria monocytogenes; S.aureus = Staphylococcus aureus, S.pneumoniae= Streptococcal pneumoniae, E.coli= Escherichia coli, H.influenzae-Haemophilus influenzae, N.meningitidis= Neisseria meningitidis, CNS= Coagulase negative Staphylococci, Mtb= Mycobacterium tuberculosis


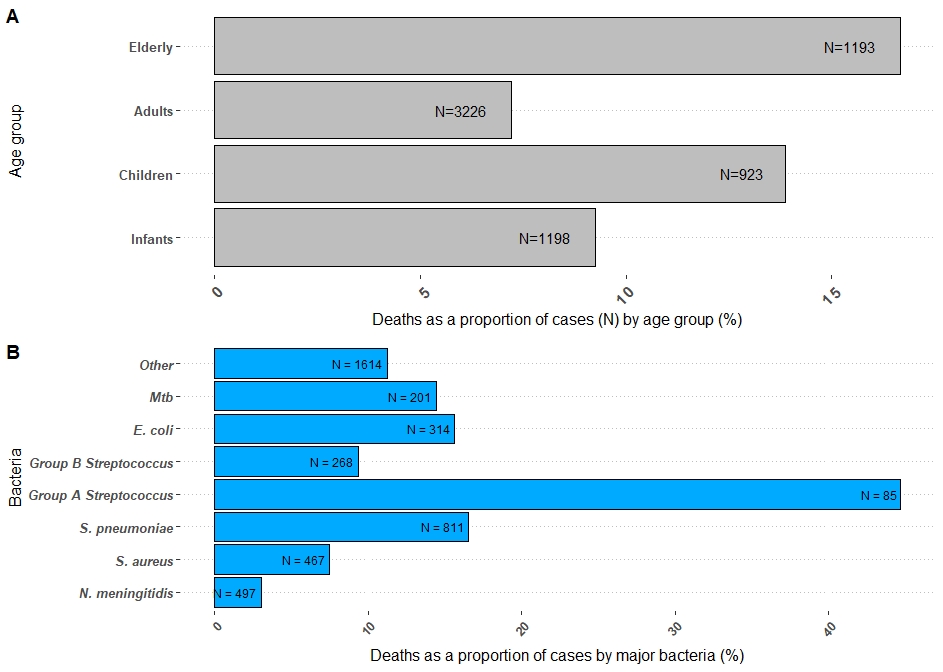


Supplementary Figure 3. Proportions of cases who died within 30 days of laboratory confirmation by age-(A) group and by pathogen (B). The numbers inside the bar-chart represent the total numbers of cases.


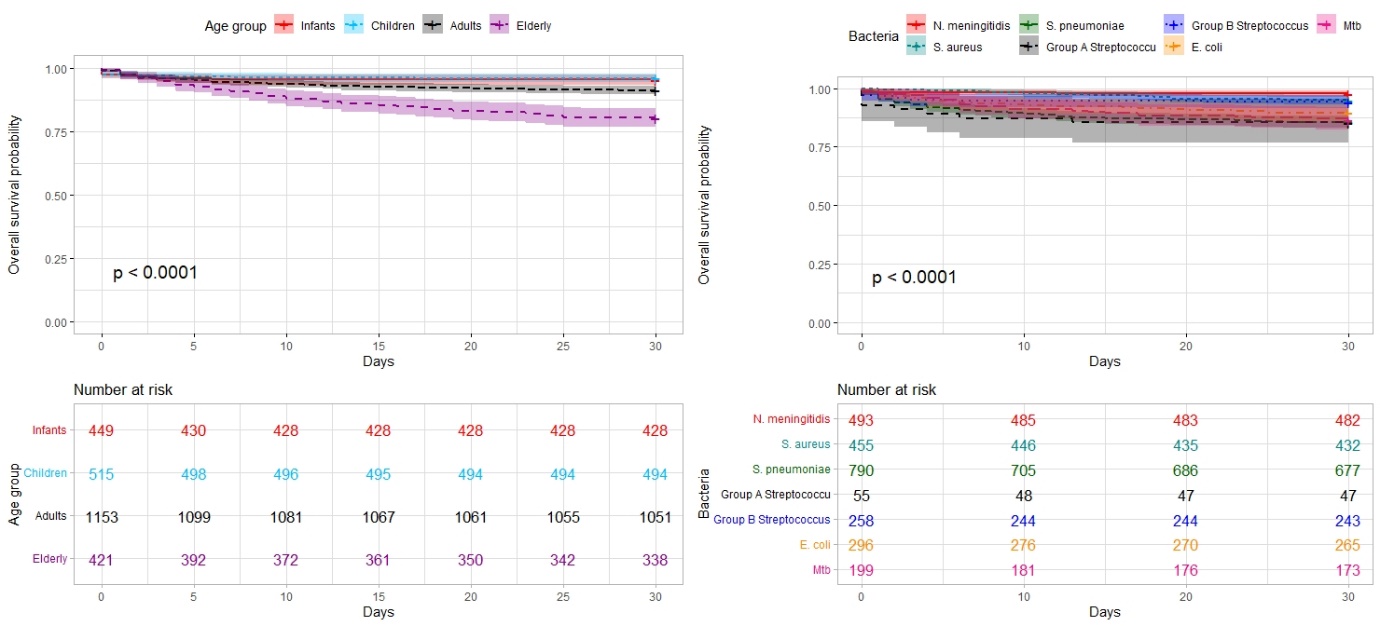


Supplementary Figure 4. Kaplan-Meier curves for survival probabilities at 30 days stratified by age group and pathogen. Follow-up period was limited to 30 days; those that did not linked to an electronic Patient Demographic Service (PDS) record to confirm status (alive/dead) and those who died after 30 days after their test date were censored. The tables below each curve summarises those at risk at 5-day intervals. Statistical significance was estimated by the log-rank calculation.
