# Supplementary material for: Trends in laboratory-confirmed bacterial meningitis (2012–2019): national observational study, England
Source: Lancet Reg Health Eur. 2023 Jul 25;32:100692. doi: 10.1016/j.lanepe.2023.100692 (PMC10393823; doi:10.1016/j.lanepe.2023.100692)
Supplement: APPENDIX [file mmc2.docx]

**Appendix**

Table of removed organisms (either as clinical relevance not known or frequency too few)

| Organism | Frequency |
| --- | --- |
| ADVENELLA SP | 1 |
| AEROCOCCUS SP | 5 |
| AEROCOCCUS URINAE | 2 |
| AEROCOCCUS VIRIDANS | 14 |
| AGROBACTERIUM SP | 1 |
| BACILLUS CEREUS | 17 |
| BACILLUS CIRCULANS | 5 |
| BACILLUS FLEXUS | 1 |
| BACILLUS LICHENIFORMIS | 7 |
| BACILLUS MEGATERIUM | 1 |
| BACILLUS OTHER NAMED | 8 |
| BACILLUS PUMILUS | 9 |
| BACILLUS SIMPLEX | 4 |
| BACILLUS SP | 181 |
| BACILLUS SUBTILIS | 3 |
| BACTEROIDES DISTASONIS | 1 |
| BACTEROIDES SP | 1 |
| BIFIDOBACTERIUM BREVE | 5 |
| BIFIDOBACTERIUM NAMED | 2 |
| BIFIDOBACTERIUM SP | 1 |
| BREVIBACTERIUM CASEI | 2 |
| BREVIBACTERIUM SP | 6 |
| CARNOBACTERIUM MALTAROMATICUM | 1 |
| CELLULOSIMICROBIUM CELLULANS | 1 |
| CLOSTRIDIUM BIFERMENTANS | 1 |
| CLOSTRIDIUM CLOSTRIDIFORME | 1 |
| CLOSTRIDIUM DIFFICILE | 42 |
| CLOSTRIDIUM DISPORICUM | 1 |
| CLOSTRIDIUM OTHER NAMED | 1 |
| CLOSTRIDIUM PERFRINGENS | 9 |
| CLOSTRIDIUM SEPTICUM | 1 |
| CLOSTRIDIUM SORDELLI | 2 |
| CLOSTRIDIUM SP | 6 |
| CLOSTRIDIUM SPOROGENES | 1 |
| CORYNEBACTERIUM ACCOLENS | 7 |
| CORYNEBACTERIUM AFERMENTANS | 1 |
| CORYNEBACTERIUM AMYCOLATUM | 8 |
| CORYNEBACTERIUM AURIMUCOSUM | 2 |
| CORYNEBACTERIUM COYLEAE | 4 |
| CORYNEBACTERIUM DIPHTHERIAE | 1 |
| CORYNEBACTERIUM IMITANS | 2 |
| CORYNEBACTERIUM JEIKEIUM (JK) | 23 |
| CORYNEBACTERIUM MINUTISSIMUM | 3 |
| CORYNEBACTERIUM MUCIFACIENS | 1 |
| CORYNEBACTERIUM OTHER NAMED | 9 |
| CORYNEBACTERIUM PROPINQUUM | 2 |
| CORYNEBACTERIUM PSEUDODIPHTHERITICUM | 8 |
| CORYNEBACTERIUM SIMULANS | 1 |
| CORYNEBACTERIUM SP | 63 |
| CORYNEBACTERIUM STRIATUM | 17 |
| CORYNEBACTERIUM UREALYTICUM | 2 |
| CORYNEBACTERIUM XEROSIS | 1 |
| CUTIBACTERIUM ACNES (PROPIONIBACTERIUM ACNES) | 747 |
| DELFTIA ACIDOVORANS (COMAMONAS ACIDOVORANS) | 2 |
| DELFTIA SP | 1 |
| DERMABACTER HOMINIS | 2 |
| DERMACOCCUS NISHINOMYAENSIS | 2 |
| DERMACOCCUS SP | 1 |
| DIPHTHEROIDS | 203 |
| DOLOSIGRANULUM PIGRUM | 1 |
| FINEGOLDIA MAGNA | 2 |
| KOCURIA KRISTINAE | 4 |
| KOCURIA RHIZOPHILA | 12 |
| KOCURIA SP | 3 |
| LACTOBACILLUS FERMENTUM | 1 |
| LACTOBACILLUS GASSERI | 1 |
| LACTOBACILLUS LACTIS | 2 |
| LACTOBACILLUS OTHER NAMED | 1 |
| LACTOBACILLUS PARACASEI | 1 |
| LACTOBACILLUS RHAMNOSUS | 5 |
| LACTOCOCCUS LACTIS | 10 |
| LACTOCOCCUS SP | 3 |
| LEUCONOSTOC CITREUM | 1 |
| LEUCONOSTOC PSEUDOMESENTEROIDES | 1 |
| LEUCONOSTOC SP | 16 |
| MASSILIA TIMONAE | 1 |
| MICROCOCCUS LUTEUS (SARCINA) | 190 |
| MICROCOCCUS OTHER NAMED | 1 |
| MICROCOCCUS SP | 84 |
| PAENIBACILLUS GLUCANOLYTICUS | 3 |
| PAENIBACILLUS SP | 6 |
| PARVIMONAS MICRA | 3 |
| PEPTONIPHILUS HAREI (PEPTOSTREPTOCOCCUS HAREI) | 1 |
| PEPTOSTREPTOCOCCUS OTHER NAMED | 2 |
| PROPIONIBACTERIUM FREUDENREICHII | 223 |
| PROPIONIBACTERIUM GRANULOSUM | 1 |
| PROPIONIBACTERIUM SP | 6 |
| RAHNELLA NAMED | 1 |
| SLACKIA EXIGUA | 1 |
| STOMATOCOCCUS MUCILAGINOSUS | 18 |
| STREPTOCOCCUS ALPHA AND NON-HAEMOLYTIC | 728 |
| Grand Total | 2790 |
